# Understanding the Psychological Impacts of Shielding From COVID‐19 for Immunocompromised Individuals

**DOI:** 10.1111/hex.70675

**Published:** 2026-04-16

**Authors:** Anna Gray, Jo Daniels, Luca Bernardi, Julie Barnett

**Affiliations:** ^1^ Department of Psychology University of Bath Bath UK; ^2^ Department of Politics University of Liverpool Liverpool UK

**Keywords:** coronavirus, COVID‐19, immunocompromised, pandemic, shielding

## Abstract

**Objectives:**

This study aimed to explore the impact of shielding amongst immunocompromised individuals following the COVID‐19 pandemic.

**Design:**

This study used a cross‐sectional exploratory qualitative design.

**Methods:**

A national survey was launched in July 2023 aiming to collect information on the lived experience of individuals who were still shielding. The final question to this study was ‘what has been the worst aspect of shielding for you?’. In total, 457 individuals (57%) responded to this question. Data were analysed using thematic analysis.

**Results:**

Three themes were identified, associated with the impact of shielding:

(1) Navigating risk: including fear of infection, distrust in institutional guidance and measures.

(2) Loss and longing: including profound isolation, grieving lost time, loss of freedom and relationships.

(3) Betrayed and forsaken: a sense of intentional abandonment by government and lack of understanding from loved ones, causing distress and mistrust.

**Conclusion:**

The findings from this study indicate that individuals who are shielding continue to experience profound difficulties that require attention. Measures must be introduced to mitigate the impact of COVID in the immunocompromised, including improving public health messaging and strategies to tackle isolation. Given the high likelihood of future pandemics, it is imperative that strategies are developed to address the needs of its most vulnerable populations.

**Patient or Public Contribution:**

Patient and public contribution has formed an integral part in the assignment of this study. The rationale and design of this study was created in partnership with Forgotten Lives UK and the National Clinical Expert Group, who represent 125 clinicians, across 4 nations and 17 medical specialities treating immunocompromised patients.

## 1 Introduction

Five years ago, in March 2020, the government introduced the first ‘lockdown’ in England, a behavioural control measure designed to limit the spread of COVID‐19 [1]. It was soon identified that individuals with compromised immune systems, for instance those who had undergone an organ transplant or who had serious lung conditions, would be at greater risk of death or severe illness if they were to be infected with the disease compared to the general population [2]. These individuals were labelled ‘clinically extremely vulnerable’ (CEV) and 4 million [3] were added to a ‘Shielded Patient List’ (SPL), where they were advised to take wider protection measures than the general population [4].

Official shielding guidance ended with the rollout of the vaccination programme in September 2021 [5]. However, individuals who are immunocompromised remain at elevated risk [6, 7], with statistics stating approximately 500,000 people in England continue to be at risk of severe outcomes if they were to be infected with COVID‐19 [8, 9], and patient advocacy group Forgotten Lives UK reporting much higher numbers of up to 1.8 million people, who continue to live restricted lives owing to their immunocompromised status [10].

Individuals who shield report a range of significant disruptions to their lives, including strained interpersonal relationships, heightened feelings of vulnerability and disability, job loss, guilt associated with missing social events and persistent anxiety about infection [11]. These cumulative stressors are likely to have contributed to the elevated symptoms of depression, anxiety and lower life satisfaction documented in this population [6, 7, 12, 13] (Daniels and Rettie 2021). The way in which the risks of COVID‐19 were and continue to be perceived has had a significant association with psychological wellbeing during the pandemic [14], as well as influencing adoption of preventative measures, whereby higher anxiety and stress levels were associated with higher engagement in hygiene behaviours [15]. Data pertaining to the efficacy of shielding is mixed, with a recent study finding that CEV individuals who shield remain at risk of infection through their healthcare contacts [16]. This highlights the challenge as to how CEV individuals can effectively keep themselves safe while accessing vital healthcare needs safely.

The Extended Parallel Process Model (EPPM) provides a useful lens to consider how emotional responses, particularly fear, interact with appraisals of risk for those that are shielding [17]. Engagement in protective behaviour is shaped by two key perceptions: the perceived severity of a threat (i.e. its potential magnitude) and perceived susceptibility (i.e. the likelihood of experiencing the threat) [17]. Given that immunocompromised individuals face an elevated risk of severe outcomes from COVID‐19, it is unsurprising that they report elevated levels of fear [18].

EPPM posits that individuals' responses to health threats are also shaped by their appraisal of efficacy. Perceived efficacy encompasses two cognitive evaluations: response efficacy, referring to beliefs about the effectiveness of protective measures such as vaccination or masking, and self‐efficacy, which reflects confidence in one's ability to enact these measures effectively, such as the ability to be adequately protected in a public space [17]. Evidence suggests that individuals with health comorbidities have diminished self‐efficacy [19, 20]. Self‐efficacy has been found to improve in the presence of social support for those receiving treatment for COVID‐19 [21], indicating that individuals who are isolated may experience heightened risk perception.

When individuals perceive a threat as severe, but feel low efficacy in managing it, they may engage in a ‘fear control’ response, involving behaviours such as avoidance or withdrawal to reduce emotional discomfort [22]. However, these responses can have implications for mental health. For example, higher levels of fear control have been identified as predictors of depression and anxiety amongst Chinese pregnant women during the COVID‐19 pandemic [23]. The avoidance associated with fear control may also contribute to social isolation, which can be detrimental to psychological wellbeing [24].

The Middle‐Range Theory of Social Isolation (MRTSI) [25] suggests that individuals with chronic conditions, such as those who are shielding, are especially vulnerable to experiencing loneliness and isolation, due to a greater need for emotional and practical support, which in this specific set of circumstances, will have risen alongside a decline in available support. A core tenet of MRTSI is that objective social disconnectedness, defined by limited social contact or engagement, amplifies subjective feelings of loneliness. Shielding entails reduced social interaction to reduce disease transmission, yet those with health comorbidities are in greater need of social support [26], leading to high risk of loneliness and isolation. A lack of belonging and stigma may precede the onset of social isolation and loneliness [25] in those who were ‘labelled’ as CEV. Furthermore, periods of social isolation may further entrench social withdrawal by reducing confidence and motivation to re‐engage socially [27], thereby perpetuating the cycle. Negative feedback loops such as this are parallel to psychological models of low mood and depression [28] and may therefore help to explain the higher rates of depression documented in this cohort [13]. Immunocompromised individuals have also reported experiencing negative medical interactions during the pandemic, feeling as though they were ‘abandoned’ by medical staff, that they ‘counted less’, and were made to feel disabled by their vulnerability to COVID‐19 [29], which was reinforced by death tolls being prefaced with the proportion of those who were already vulnerable [11].

There are clear global and national drivers in understanding the experiences of those who continue to shield in the wake of pandemics. The risk of sudden viral outbreaks has increased over recent years [30], making it timely to learn from the COVID‐19 pandemic and consider global preparedness for future pandemics [31]. The World Health Organisation (WHO) has created a ‘Preparedness and Resilience for Emerging Threats Initiative’ and launched a ‘Call to Action’ to help consider learnings from the COVID‐19 pandemic and assist planning for future threats [32]. In the United Kingdom, Exercise Pegasus, a national pandemic preparedness simulation, has been launched in 2025 to refine the country's pandemic response [33]. Understanding the lived experience of those who are most vulnerable to the effects of the pandemic will be vital in learning how to shape future pandemic responses.

In the United Kingdom, the COVID‐19 enquiry [34] highlighted failures in safeguarding the most vulnerable in society, prompting emphasis on understanding the mental health needs of this population. This concern has been echoed in parliament, through the establishment of an All‐Party Parliamentary Group (APPG) for Vulnerable Groups to Pandemics [35]. The APPG has since proposed recommendations including prioritising and streamlining access to antivirals and COVID‐19 therapies, which underscores the importance of an equitable healthcare response in future pandemic scenarios.

We therefore aim to answer the research question: ‘What insights can be gained into the psychological impact of shielding among immunocompromised individuals, and how might these inform more effective protection strategies for this population in future pandemics?’.

## 2 Methodology and Design

### 2.1 Study Design and Data Collection

Between July and September 2023, a national mixed‐methods survey was launched to collect data on the experiences of people who were immunocompromised and still shielding due to COVID‐19, compared to the general population [36]. The analysis in this paper is derived from the qualitative data only, collected in the wider mixed‐methods survey.

The study was advertised by Forgotten Lives UK, a patient‐led campaigning group, on their website and associated social media accounts.

The quantitative arm of the survey (not analysed in the current study) began with a series of questions on a Likert scale, designed to measure the following: COVID‐19 worries, mental health and wellbeing, and political engagement. Results from the quantitative survey identified that, in this shielding sample, COVID‐19 worry factors are associated with symptoms of depression and, in turn, to trust in government and satisfaction with democracy. Symptoms of depression are also found to be associated with COVID‐19 stress and, in turn, to political attention and internal political efficacy [37]. The noted implications of this quantitative arm of the study relate to addressing psychological vulnerability, and the importance of recognising the political views and needs of this group.

The current study focuses on the analysis of the survey's qualitative free‐text question: ‘What has been the worst aspect of shielding for you during the COVID‐19 (coronavirus) pandemic?’. The design of this question is broad and open‐ended to elicit responses based on subjective lived experience of shielding. By focusing on the ‘worst aspect’ participants are encouraged to share the most salient impacts of shielding for them.

### 2.2 Participants and Procedure

For the qualitative arm of this research, participants were excluded if they did not answer ‘Myself Only’ or ‘Myself and Others’ in response to a question asking who they were shielding. Eligibility for the wider study required participants to be aged over 18 and residing in the United Kingdom.

These inclusion criteria were deemed more appropriate than a strict requirement of CEV status, given that the ‘SPL’ cataloguing CEV individuals has been closed since 2021 [5]. Overly strict criteria regarding participants' shielding or immunocompromised status were avoided to include all those that continue to experience the lasting of effects of shielding.

A total of 457 individuals (57%) meeting the inclusion criteria responded to the qualitative free‐text survey question. In total, 36 participants were excluded as they were ineligible. The demographics of respondents can be found in Table 1. Ethical approval for this study was granted by the University of Liverpool and the University of Bath. All identifiable data were removed and anonymised.

**Table 1:** Participant characteristics.

Characteristic	Value (*n*, %)
Age (mean, standard deviation, range)	57.2 (11.99; 24–99)
Ethnicity	
English/Welsh/Scottish/Northern Irish/British	395 (85.99%)
Any other White background	21 (4.59%)
Prefer not to say	8 (1.75%)
Irish	8 (1.75%)
Any other mixed/multiple ethnic background	7 (1.53%)
Indian	5 (1.09%)
Any other ethnic group	3 (0.66%)
White and Asian	3 (0.66%)
African	2 (0.44%)
Any other Asian background	1 (0.22%)
Caribbean	1 (0.22%)
Chinese	1 (0.22%)
White and Black African	1(0.22%)
White and Black Caribbean	1 (0.22%)
Gender	
Female	345 (75.05%)
Male	109 (23.85%)
Other	3 (0.66%)
Who is shielding	
Myself	298 (65.2%)
Myself and others	159 (34.79%)

### 2.3 Data Analysis

Answers to the question were analysed using Reflexive Thematic Analysis (TA) [38]. This involved six main steps: data familiarisation, preliminary coding, identifying themes, reviewing themes, defining and naming themes, and producing the report. Codes and associated themes were identified based on their relevance to the ongoing impact of shielding on individuals.

A. G. conducted this process independently using an inductive, bottom‐up approach. A critical realist epistemological stance was taken. This was deemed appropriate as, while we expected the accounts of our participants to tell us something about reality, this reality was that of the world view of both the interviewer and interviewee [39]. This meant that we were not only exploring the ongoing impact of shielding but aiming to contextualise these experiences in deeper structural and societal mechanisms that may be shaping their experiences. EPPM and MRTSI were used as conceptual lenses to support with data analysis and while they were not used to drive coding or theme development, they allowed for a deeper understanding and interpretation of participant accounts.

Following data familiarisation, A. G. analysed the data and assigned codes to participant descriptions of their experiences of shielding, with 58 codes being generated. In line with the values embedded in reflexive thematic analysis, codes were defined based on their conceptual meaning [40]. As such, concepts such as data saturation and theme redundancy were not utilised, as themes were generated through interpretive and reflexive engagement with the data rather than adherence to procedural criteria.

Throughout this process, A. G. regularly consulted with J. B. to sense‐check coding decisions and share and invite reflections on the developing themes. A. G. documented observations, reflexive notes and initial identification of themes throughout as a basis for discussion with the wider research team (J. B., J. D. and L. B.), who challenged justifications for initial themes and provided alternative perspectives. Personal stances were reflected on, with each team member having different experiences in the field, alongside unique experiences of being impacted by the pandemic, which may have influenced interaction with the data. Following continuous analysis and adjustment, three final themes were agreed by all team members.

## 3 Results

Three overarching themes were identified, labelled: (1) Navigating risk, (2) Loss and longing and (3) Betrayed and forsaken. Three themes were deemed appropriate, as they reflect a concise narrative of the conceptual and shared meaning between the many topics raised by participants.

### 3.1 Navigating Risk

Individuals who were required to shield are, by definition, vulnerable to the acute health impacts resulting from COVID‐19 infection. Many of these individuals will have already experienced an altered awareness of their own vulnerability associated with their pre‐existing health conditions. Participants described how they felt the immense risk associated with catching COVID‐19. The assessment of this risk was heightened, owing to perceptions of an extremely negative outcome if they, or their family members were to catch the disease:

The utter desperation and fear I have felt since March 2020. The fear that I would be seriously ill or die from Covid has been unbearable. Every time I thought that help was coming for me and the CEV community my hopes were cruelly dashed.

Female, 62

Accounts such as these reflect how, beyond the backdrop of shielding, individuals were navigating an ongoing state of fear and threat. While shielding precautions were acknowledged to reduce the possibility of these feared outcomes and thereby reduce threat, for many this was juxtaposed with a sense that those around them were either unaware or unwilling to acknowledge this immense risk and therefore take appropriate precautions to keep others safe:

I have to go to numerous medical appointments with doctors who know my medical history and vulnerabilities and they still don't mask when they see me wearing an FPP2‐why should I have any trust in any systems at this point.

Female, 37

Trying to get my husband to understand that he shouldn't mix with others. He caught Covid. Luckily I didn't. With his behaviour I don't know how I didn't.

Female, 72

As exemplified in these quotes, the fear associated with catching the disease is influenced by the actions/inaction of both institutions and individuals, which appears to be associated with a sense of distrust, while also highlighting how much of the presenting risk felt outside of their own control. This was made particularly pertinent by participants who described their increase in distress when lockdown measures were lifted. This exemplifies the fear associated with one's safety lying in the hands of others:

I didn't find shielding so bad (apart from the worry of getting sick of course). I've found it's much much worse now there are zero mitigations in place.

Female, 37

Shielding made me feel safe. The lack of provision in terms of ventilation in public spaces, schools and workplaces means that life is now more stressful.

Female, 41

These quotes highlight how collective societal measures of shielding felt reassuring, however, their removal left individuals feeling vulnerable and exposed. In the face of the withdrawal of public health measures and intensified threat, the management of risk shifted towards becoming a personal responsibility, whereby participants described taking personal actions for self‐protection. These actions were far reaching and often came at significant personal and financial sacrifices:

Last year I had to find the drug (Evusheld) for myself because I felt it might help. I have also spent a great deal of money on HEPA filter machines to enable me to go visit places such as family and cafes.

Female, 57

The lack of mitigations in schools and the lack of understanding about concerns about passing on infections has made me distrust the education system and we are now paying for our daughter to attend an online school.

Female, 48

The fact that individuals were willing to take such far‐reaching significant personal and financial sacrifices, highlights the high level of threat individuals felt from COVID‐19. However, even in the context of taking personal responsibility over risk management, there was still a high degree of uncertainty as to how to contain the risks, which was connected to a lack of clear official guidance:

Lack of new support and guidance in recent years as risk still extremely high for us who are immune compromised.

Female, 51

Even some immunocompromised people are unaware of the guidance which applies to them as the Government have never specifically highlighted the detailed guidance specifically to the immunocompromised.

Male, 61

These findings illuminate the challenges of risk management, in the context of the absence of clear and trusted guidance on what good and effective risk management would look like.

### 3.2 Loss and Longing

Longing is defined as a strong desire for something unattainable or distant [41] and is deeply rooted in the human experience. The responses from immunocompromised participants in the current study describe the profound changes in their lives that shielding necessitated, which are likely to have compounded losses already experienced in relation to their health conditions. This was particularly pertinent in the context of social isolation, with many participants describing the pain of being apart from loved ones:

The worst part has not been able to spend time with my mum as she was dying from dementia… Before shielding I used to see my mum every other day and help care for her… Missing weddings, birthdays, funerals.

Female, 55

The loneliness. I was unable to work due to the risk, and I live alone. I no longer feel I can join groups or go out socially unless I do so outdoors in a small group. I worry if someone needs to come into the house for repairs etc… I can no longer mix with people to make friends, so my life has become very isolated and I rely mostly on my daughter for company. It can be very depressing.

Female, 67

These accounts convey how shielding meant that individuals lost moments with loved ones during some of life's most significant events that could never be retrieved. In addition to loss being described in the context of interpersonal relations, it was also described on an intrapersonal level, whereby individuals mourned the loss of the freedoms and a lifestyle they had once enjoyed. This was accompanied by a sense of life losing almost all meaning and purpose:

Loss of my business and my employees, consequential loss of income, loss of any social interaction and quality of life.

Male, 59

Loss of access to normal patterns of life – no shopping, socialising, trips out, eating out, visiting friends and family, going into work – I have been a prisoner in my own home for over 3 years and this government couldn't care less.

Female, 53

At a more existential level, these losses were described in the context of losing a sense of ‘self’, and a fundamental shift in identity, because of shielding. Resultantly, there was a sense that even if life were to return to ‘normal’ on a practical level, things would never be the same:

The pandemic in a way has changed my personality and interaction with people, which has put a massive strain on my marriage!

Male, 53

Distinct changes in my personality. I am profoundly disappointed with people, feel betrayed and all trust is broken.

Female, 65

These accounts reflect how shielding not only disrupted daily life, but was a rupture of personal identity‐distress was not only situational, but created more enduring changes in self‐concept. Amongst such a fundamental shift, questions may arise about what the future holds. While longing towards the future can be seen as a motivating force for change, imagining that future can feel murky and lacking in clarity when there is so much uncertainty:

It makes me feel that there is no light at the end of the tunnel.

Female, 55

It feels like it's never going to end for those at high risk.

Female, 54

Such reflections on the value of time were also discussed in the context of lost, even stolen time, that could never be retrieved:

I have lost 3 years and cannot get them back because I am now more disabled and more limited in what I can do compared with a trip just before COVID.

Female, 60

Taken together, these findings illuminate a sense of irretrievable loss and a feeling of being ‘stuck’ with a lack of clarity on how to move forwards, leaving individuals in an ongoing state of mourning.

### 3.3 Betrayed and Forsaken

A sense of betrayal was frequently referred to by participants. In the most distressing accounts, this betrayal was felt to be an intentional act, rooted in a feeling that the lives of immunocompromised individuals are viewed as less valuable, almost disposable. Such betrayal was often attributed to the actions of government:

‘Let the bodies pile high’ was directly aimed at us.^1^

Female, 60

Government policy of living with Covid is undemocratic and does not follow the philosophy of protecting the health of the population…This policy is about allowing vulnerable people to die.

Male, 77

The gaslighting from the government and society that COVID is no longer a problem. That my genuine, valid concern for the health of myself and others is disregarded as me being ‘anxious’.

Female, 35

I feel that I have been totally forgotten by the government I voted for. By not finding Evusheld and reducing treatments available, by reducing rules in hospital by which to reduce COVID, I feel that they don't care about the forgotten cev.

Female, 57

The above quotes indicate a sense of the profound betrayal felt from those in power, which felt calculated and intentional. Betrayal was also described by participants whereby they described feeling deeply misunderstood by those around them who were not immunocompromised. This was particularly challenging when this lack of understanding came from loved ones:

People singling me out as different or weird or unworthy because I'm doing what I know is right for me and for others. People telling me I'm not educated enough to understand research on Covid… The shielding and missing out on important life events and my hobbies and interests is actually the easy part. The lack of understanding about that is very hard to deal with.

Female, 27

Knowing I am right to carefully shield but feeling more and more isolated, being perceived as neurotic and unacknowledged, as COVID continues.

Female, 59

My life doesn't matter, and my friends have abandoned me because they don't understand what the risks are to me if I get COVID.

Female, 70

What seems clear from the above quotes is that it was not only the physical separation that led to a sense of isolation for participants, but also a sense of emotional isolation in being so misunderstood. Furthermore, a lack of protective action from others not only increased a sense of fear for the immunocompromised but also increased a sense of social isolation:

Feeling of being discarded and discounted by society.

Female, 66

Feeling invisible now the rest of society has decided COVID is over.

Female, 46

Now society looks at me with contempt, and that I am causing problems by continuing to take precautions.

Male, 25

This feeling of being ‘singled out’, is reflected in participants frequently referencing their own position against that of ‘society’ as a whole. Such references to society indicate a sense that individuals no longer feel as though they form part of it.

## 4 Discussion

The identified themes highlight how the impact of shielding on the clinically vulnerable is multifaceted. The risks and potential consequences of catching COVID‐19 were associated with fear and anxiety, which was a motivating force to engage in infection‐control measures such as social distancing. Enacting shielding came at huge personal sacrifice including loss of employment, hobbies and social isolation. These losses were associated with a sense of sadness, a longing for a previous life and a shift in identity that felt permanent. Perhaps most distressing was an overpowering sense of abandonment and neglect by government, healthcare providers, loved ones and wider society. This was associated with a sense of betrayal and anger, with little sense of how trust could be regained. In interpreting these findings, it is crucial to be considerate of the fact that participants self‐identified as immunocompromised and a strict clinical definition was not used. As such, these findings are rooted in the meaning of participants' lived experiences. This has provided an important and unique window into how individuals make sense of risk and vulnerability and is therefore equally consequential when compared to formal clinical definitions, in understanding how to support this group of people.

### 4.1 Navigating Risk

This theme encapsulates the immense threat that participants felt which was addressed by shielding. Participants described fear in the context of their own vulnerability to the disease and its potential outcomes, distrust in the actions of other individuals and institutions, amidst unclear official guidance in how to limit the threat of the disease. These findings are consistent with the EPPM theory, which states that high perceived severity of the disease will trigger a risk response, even in the face of reduced likelihood [17]. This helps to explain why individuals continue to shield, even after COVID‐19 became less prevalent in the population [43]. Regardless of the levels of the disease in the population, the risk associated with catching COVID‐19 for those with underlying medical conditions is still high [44]. Greater public and professional understanding of the dynamics of risk perception may enable greater empathy and understanding as to why immunocompromised individuals continue to shield.

This theme also captures a strong sense that those navigating risk by shielding in part attribute this to public health measures falling significantly short in offering protection from the risks of COVID‐19. Furthermore, advice from media and government communication channels were reported to be inconsistent, in contrast to the necessary reliance on the media as a channel of communication to convey accurate risk‐based information [45]. Given this attenuated response efficacy, individuals are required to rely on their self‐efficacy by engaging in their own risk management behaviours [17]. Given the uncertainty as to how well these will afford protection from infection, these are unlikely to dissipate stress and tension [22] and this, in part at least accounts for the distress recounted by our participants.

The high levels of fear and distress by participants is consistent with research by Rettie and Daniels [46], who found that immunocompromised individuals who were a member of a vulnerable group during the pandemic, were significantly more likely to reach the threshold for health anxiety or generalised anxiety than the general population. This was attributed to the increased threat of the disease, necessitating infection‐control measures such as hyper‐vigilance and avoiding contamination—behaviours which may indirectly contribute to health‐related anxiety in those who are already vulnerable [47]. Interestingly, they did not find that vulnerable individuals were less tolerant to uncertainty than the public. An explanation for this may be that individuals who are immunocompromised are likely to have navigated many significant health‐related challenges in their lives prior to the pandemic and may be more resilient to the effects of uncertainty [46].

The EPPM is able to account for the high level of reported distress, as it would suggest that immunocompromised individuals who continue to shield are experiencing high perceived threat and low perceived efficacy in response to COVID‐19, leading to subsequent chronic fear control responses and high fear arousal [20] followed by subsequent behavioural avoidance, which psychological models suggest will serve to contribute to the maintenance of anxiety or depression through negative feedback loops [48].

### 4.2 Loss and Longing

Individuals with long‐term health conditions, many of whom have undergone significant transitions in their lives, may be more likely to experience a sense of longing for their previous life, for a relief of suffering in their current situation, and longing for clarity for the future [41]. This theme captures a sense of profound loss that our participants experienced in relation to shielding, that may have amplified losses already experienced in relation to their health conditions. This loss was far reaching: it heightened social isolation and loneliness, led to loss of opportunity and undermined future hopes.

These findings are consistent with the MRTSI, stating that individuals with chronic illness are already at risk of loneliness owing to factors such as pain management, fatigue and unemployment [25]. Immunocompromised individuals therefore represent a group of people at risk of extreme isolation owing to the dual impact of their health conditions and shielding. The clinical implications for this are significant, since isolation has been found to consistently be a predictor of poor mental and physical health outcomes [49].

This theme captures how participants shared experiences of losing the opportunity to engage in important aspects of their lives, such as work or travel. This was accompanied by a shift in identity, whereby participants described a ‘changed’ sense of self, which felt permanent. This may further exacerbate feelings of loneliness and isolation, as the MRTSI would suggest that a lack of belonging and low‐self‐esteem are contributing factors to isolation [50]. Psychological theories of depression would suggest that such losses could increase vulnerability, as factors such as social withdrawal and negative thoughts about the self and others, may reinforce the negative view the individual holds of themselves [51]. Harkin et al. [52] therefore suggest that it is not enough to simply relax social rules to aid the recovery from loneliness and isolation. Instead, individuals should be offered opportunities to restore or regain identities.

### 4.3 Betrayed and Forsaken

Participants descriptions of betrayal are harrowing and reflect the stark psychological impact on many individuals who continue to shield. What seems evident from these extracts is that this betrayal was felt to be an intentional act, whereby individuals described feeling as though others felt that their lives were less valuable. The MRTSI posits stigma as a primary precipitating factor to social isolation, by undermining social interaction and predisposing individuals to loneliness, leading to further withdrawal [25]. The concept of betrayal‐based moral injury may further our understanding of the role of stigma in reinforcing social isolation. Moral injury is a term used to describe the distress that can arise when the actions of individuals or institutions violates a personal moral code [53] and, more specifically, betrayal‐based moral injury occurs when the moral betrayal is conducted by those in a trustworthy position [54]. Taken together, these theories can explain how the pandemic did not only lead to a sense of profound loneliness amongst the CEV community, but also a deep‐rooted perception that society devalued their lives.

Betrayed and forsaken reflects how participants' trust in institutions, including the government and NHS, had been severely undermined. The EPPM would suggest that when institutions are perceived to not be acting in good faith, the trust in their mechanisms to keep individuals safe from harm will be undermined, leading to a reduced ‘perceived efficacy’ in response to the threat of COVID‐19. This low perceived efficacy will lead individuals to engage in fear‐control responses, which subsequently increases anxiety [17]. The EPPM is therefore able to explain how feelings of betrayal may be contributing to the increased clinical anxiety that is evident in this current research and documented in the literature [46].

Given that this participant group was recruited from the charity for Forgotten Lives UK and associated groups, this theme may capture the experiences of individuals who may have perceived particularly significant betrayals and may therefore be more like to engage in research studies to raise awareness and advocate for others in a similar situation, potentially contributing to participation bias [55]. In total, 43% of individuals who completed the quantitative arm of the survey did not respond to the qualitative free‐text question, who may not have shared similar difficulties. Regardless of this, the narratives captured here illuminate perspectives on the distress experienced by a significant number of vulnerable individuals, who report feeling ignored and inadequately protected or supported.

### 4.4 Implications for Public Policy and Clinical Practice

Drawing on our findings, we make several recommendations for public policy and clinical practice specifically pertaining to the identified themes.

Immunocompromised individuals would benefit from measures that would support them to navigate risk. This can be addressed at both a public policy and clinical practice level. At a system level, those deemed clinically vulnerable could be added to the NHS England Risk Register, meaning that immunocompromised individuals would be automatically flagged and prioritised to receive practical support to reduce risk (e.g., food boxes), and receive preventative treatments and vaccinations in the event of a future infectious disease outbreak [37, 56]. However, we note that the evidence base for these prophylactic medications is still emerging [57] with more research urgently needed, and there is no clear policy guidance on how prophylactic uptake would affect shielding programmes and guidance [57]. Both would need to be developed in tandem.

Risk navigation should also be addressed at an organisational level, given previous research indicating that CEV individuals who shield remain at risk of infection through their healthcare contacts [16]. This might involve a multi‐pronged approach, such as increased public health messaging on likely risk of infection in order to inform decision‐making around health‐related behaviours, additional precautionary measures taken by healthcare staff and further practical adaptation of social distancing and transition to online clinics, for example. Navigating risk is also about risk tolerance [58] which could afford the opportunity to mitigate against social isolation and access to healthcare, through a risk and benefit analysis. This could form part of intervention at an individual level, with the provision of community teams to support those most isolated and affected. Risk perception is a core part of CBT for health‐related anxiety [59], which is widely available on the NHS and could be adapted to support those who are shielding to navigate risk where wellbeing is profoundly affected.

In relation to loss and longing, this again would benefit from a multi‐level intervention. At the system level, it is imperative that the Government ensure a future infectious disease outbreak is well prepared for and controlled early on, to avoid unnecessary isolation and avoidable loss. This would need to include a clear plan of interventions to support those who are clinical vulnerable (e.g., vaccination and treatment programme), protocols and processes around continuation of healthcare, and the provision of psychological support for those who are profoundly affected due to isolation. This would need to be clearly set out by Government, but delivered at an organisational and community level to meet local need.

With regards to our final theme of being betrayed and forgotten, also borne out in the quantitative sibling paper [37], there is a need for increased trust and moral repair between government institutions and the immunocompromised community. Research suggests that the repair of betrayal rests on the degree to which the ‘perpetrator’ can acknowledge and take accountability for their actions, and seek to learn from it [60]. There is an opportunity to do this through the COVID‐Enquiry and while parliament have documented criticism over the handling of the shielding programme, to date there has been no formal apology to the clinically vulnerable community [61].

The practical recommendations set out here, such as utilising the risk register, provision of appropriate pandemic preparedness plans that are tailored to the need of the clinically vulnerable (clear communication, tailored guidance), investment in resources (healthcare access, shielding food boxes), will go some way to repair or at least mitigate against future damage of trust. However, further proactive steps are required, such as greater involvement of immunocompromised individuals in pandemic planning in line with the government's own involvement strategy [62], commitment to clear and consistent information to dispel uncertainty and clear accountable leadership within government. Designating a minister to specifically oversee the needs of vulnerable groups was an ask of the government in a report to parliament in 2023 [36], however, no progress has been made on this to date.

We have made recommendations here that span system, organisation, community and individual level, however, for any of these recommendations to be implemented, change needs to take place at government level first. By taking steps towards accountability and lessons learned, trust will begin to build, and the process of preparing to navigate risk in partnership with those are most vulnerable in society will commence on a more even footing.

### 4.5 Limitations

Participants were not required to meet a strict definition of immunocompromised or shielding status in this study. This could potentially limit the precision by which the research question was answered, as it is not possible to consider factors that may potentially distinguish between participants, such as the duration and extent of shielding behaviours, or level of clinical vulnerability. However, it is clear from the current research that the motivation to shield extends beyond official guidance or formal classification of vulnerability. We are confident, therefore, that the inclusive nature of this research provides a comprehensive account into the realities of the impact of shielding on immunocompromised individuals.

Data were collected via an online survey and the technological requirements may have been a barrier to some individuals taking part, however, uptake was significant, and online surveys offer the potential to generate rich and nuanced insights into a given topic, particularly through the collection of a large quantity of qualitative data while offering a degree of anonymity. They are also advantageous in terms of their practicality and accessibility when compared to more time‐intensive methods such as interviews [63]. This flexibility is particularly beneficial for individuals who are shielding, as they may face health‐related challenges or caregiving responsibilities that limit their capacity to engage in long‐form interviews.

This study recruited participants via shielding social media networks, including members of the charity Forgotten Lives UK. Individuals who are engaged in these networks may be more likely to have ongoing concerns about shielding, be more politically motivated and engaged, and feel particularly unrepresented by current public policy [36]. Therefore, this study may represent those who have had particularly difficult experiences and may not capture the experiences of those who have been less affected. Future research aimed at understanding experiences across the spectrum could provide valuable information on the factors that shape these outcomes.

## 5 Conclusion

The aim of this study was to gain insights into the psychological impact of shielding among immunocompromised individuals, and how these can inform more effective protection strategies for this population in future pandemics. In doing so, providing a voice to the most vulnerable in society during the pandemic. Responses from 457 individuals reflected that shielding has led to a sense of profound and irretrievable loss and loneliness amongst the CEV community, an intense fear of COVID‐19 with little sense of how to effectively reduce this risk, alongside a notable and deep‐rooted perception that society devalued their lives. Key implications include increasing measures to reduce the severity of the impact of contagions in vulnerable groups, improving public health messaging and involving immunocompromised individuals in pandemic planning, to develop policies that are better informed and built on our understanding of the risks associated with shielding. Given the high likelihood of pandemics and further infectious outbreaks in the future, it is imperative that strategies are put in place to address the needs of our most vulnerable populations.

## Author Contributions

**Anna Gray:** writing – original draft, formal analysis, visualisation, methodology. **Jo Daniels:** conceptualisation, investigation, methodology, writing – review and editing, supervision. **Luca Bernard:** conceptualisation, investigation, methodology, writing – review and editing. **Julie Barnett:** writing – review and editing, formal analysis, supervision.

## Funding

The authors received no specific funding for this work.

## Ethics Statement

Ethical approval for this study was granted by the University of Liverpool and the University of Bath.

## Conflicts of Interest

The authors declare no conflicts of interest.

## Data Availability

The data that support the findings of this study are available from the corresponding author upon reasonable request.

## Endnotes

1 ‘Let the bodies pile high’ is a phrase that Dominic Cummings (former Advisor to Boris Johnson) alleged that Boris Johnson used during a discussion over whether to enforce another lockdown in Britain [42] https://www.reuters.com/world/uk/uk-pms-former-adviser-confirms-johnson-said-let-bodies-pile-high-2021-05-26/).
